# Early Diagnostics of Freemartinism in Polish Holstein-Friesian Female Calves

**DOI:** 10.3390/ani9110971

**Published:** 2019-11-14

**Authors:** Anna Kozubska-Sobocińska, Grzegorz Smołucha, Barbara Danielak-Czech

**Affiliations:** Department of Animal Molecular Biology, National Research Institute of Animal Production, Krakowska 1, 32-083 Balice, Poland; anna.sobocinska@izoo.krakow.pl (A.K.-S.); barbara.czech@izoo.krakow.pl (B.D.-C.)

**Keywords:** cytomolecular diagnostics, freemartinism, Polish Holstein-Friesian breed

## Abstract

**Simple Summary:**

Freemartinism is the most common type of gender developmental disorder, resulting in infertility of heifers from multiple-sex twin pregnancies. The frequency of this syndrome is related to the frequency of multiple pregnancies, the number of which has significantly increased in dairy cattle populations (HF). Therefore, rapid diagnostics is necessary to enable early elimination of heifers with freemartinism from breeding. The aim of the study was to compare and identify the best method for early identification of freemartinism. The use of cytogenetic and molecular methods (PCR, short tandem repeats (STRs), real-time PCR) allowed us to conclude that molecular methods are more effective and guarantee fast and precise diagnosis. An additional advantage of molecular methods is the easy way to collect test material, which can be frozen, unlike blood samples for cytogenetic analysis, which must be fresh and delivered within 24 h to the laboratory, which generates further costs.

**Abstract:**

Freemartinism in females born from heterosexual multiple pregnancies is characterized by the presence of XX/XY cell lines due to the formation of a shared blood system by anastomoses between fetal membranes of co–twins and leads to disturbed development of the reproductive system, including infertility. The aim of this study was to estimate the most precise and effective diagnostic method, especially useful for early identification of freemartinism in young female calves. The cytomolecular evaluation results of 24 Holstein-Friesian heifers from heterosexual twins was verified by molecular techniques: PCR, short tandem repeats (STRs), and relative quantitative PCR. The molecular analyses have been found to be a more efficient testing strategy, with a higher diagnostic success rate than karyotype evaluation. In 21 heifers, leucocyte chimerism determined by the 60, XX/60, XY karyotype was revealed—the proportion of the 60, XY male cell line in individual animals was in the range of 4–66%. In three cases, a normal karyotype 60, XX was identified, which indicates that anastomoses did not occur in 12.5% of studied twins and suggests that these potentially fertile heifers can be qualified for further breeding. The precise and early identification of freemartinism can be the basis for guidelines and selection recommendations concerning the reproductive performance of heifers born from heterosexual multiple pregnancies.

## 1. Introduction

Freemartinism occurs in females born from twins or multiple pregnancies carrying fetuses of a different gender. In most bovine heterosexual twin females, it leads to disturbed development of the reproductive system and infertility. Freemartins are characterized by the presence of XX/XY cell lines (leukocyte chimerism) due to the formation of a shared blood system through anastomoses or vascular connections between fetal membranes of co-twins before the sexual differentiation of the fetuses. Since gonadal differentiation begins several days earlier in males than in females, it can be hypothesized that sex-determining hormones from the developing male gonad would be transported to the female fetus, potentially suppressing the reproductive organ development of the female [1,2].

Many years of research have shown that 80–95%, and even 97%, of heifers from heterosexual twin pregnancies have leukocyte chimerism and freemartinism [3,4,5,6]. The remaining 3–20% (or, as other authors state, about 10%) of females develop correctly, presumably because the placental anastomoses fail to fuse or the fusion occurs, following the critical period of reproductive organ differentiation. Taking into account the fact that frequency of multiple pregnancies in cattle is recently rising significantly (up to 20% in dairy cattle), it can be assumed that the number of fertile heterosexual twin females is also increasing [6,7,8]. Therefore, early diagnosis of normal females from heterosexual multiple pregnancies is necessary to prevent the elimination of valuable heifers from breeding. The precise cytomolecular diagnostics of freemartinism may provide a basis for formulating accurate guidelines and selection recommendations concerning the use for reproduction of heifers with XX/XY karyotype.

The purpose of this paper is to pinpoint the most precise and effective diagnostic method, especially useful for early identification of freemartinism in newborn calves.

## 2. Materials and Methods

### 2.1. Animals

Cytogenetic and molecular studies were carried out on 24 Holstein-Friesian heifers originating from heterosexual twins between the age of three days to one year. The biological material was gained from a local vet for routine testing, so no Local Research Ethics Committee permission was needed for this study.

### 2.2. Cytogenetic Diagnostics

Cytogenetic analyses were performed on Giemsa-stained preparations obtained after the 72-h lymphocyte culture. At least 250 metaphase spreads per animal studied were examined, and the proportion of the XX and XY cells were estimated. In case of difficulties concerning equivocal identification of sex chromosomes, cytogenetic diagnostics were extended by applying the FISH (fluorescent in situ hybridization) technique with the use of Bovine IDetect Chr Y Point Probe Red- (Cambio nr. kat.: IDBR1059 Cambridge, United Kingdom).

### 2.3. Molecular Genetic Methods

The cytogenetic evaluation results of all 24 heifers under study was verified by molecular analysis: PCR, short tandem repeats (STRs), and relative quantitative PCR.

DNA isolation was performed using the A&A Biotechnology Sherlock AX reagent kit (Gdynia, Poland), according to the protocols provided by the manufacturer.

A fragment of the *SRY* gene (440 bp) was amplified by PCR using the primers shown in Table 1. The PCR reaction was performed in 25 μL volume containing: 11 μL of PCR-grade water, 2.5 μL of PCR buffer with 15 mM MgCl_2_ (QIAGEN, Hilden, Germany), 5 μL of Q-Solution (5x;QIAGEN), 3 μL of 10 mM dNTPs (Thermo Fisher Scientific, Applied Biosystems, Waltham, MA, USA), 0.25 μL of primer mix (each 100 pmol/μL) (Table 1) [9], 0.25 μL of HotStartTaq DNA polymerase (5 U/μL QIAGEN), and 2 μL of DNA isolate. The PCR thermal program was as follows: 15 min of the initial activation step at 95 °C (polymerase is activated by this heating step), 35 cycles of denaturation at 95 °C for 30 s, annealing at 59 °C for 50 s, and primer extension at 72 °C for 75 s. The final extension was conducted at 72 °C for 10 min.

Amplification of the *AMEL* gene fragment was carried out with the use of primers (Table 1) [9,10,11,12,13,14,15,16,17,18]. Two fragments of the *AMEL* gene were amplified: One 280 bp-length characteristic for the X chromosome and a second (217 bp) characteristic for the Y chromosome. PCR reaction conditions were similar to those for the *SRY* gene.

The PCR products were separated by gel electrophoresis, using 2% agarose gel.

STR analysis was performed using The International Panel of Microsatellites for Cattle Parentage Testing, comprising 12 microsatellites (BM1818, BM1824, BM2113, ETH10, ETH225, ETH3, INRA023, SPS115, TGLA122, TGLA126, TGLA227, and TGLA53) (ISAG—International Society for Animal Genetics). Furthermore, in two cases, it was necessary to apply additional microsatellites (AGLA293, BM2830, CSRM60, CSSM66, HUIJ177, ILSTS065, INRA72, INRA 92, INRA 222).

Microsatellite analysis was performed on DNA isolated from blood samples of 24 heifers previously tested for *SRY* and *AMEL* genes. Microsatellites were amplified using the Type-it Microsatellite PCR kit (Qiagen) in multiplex reactions, according to the manufacturer’s recommendations. The fluorescent-labeled PCR products were submitted to fragment analysis by capillary electrophoresis, with an automated sequencer ABI PRISM 3130xl (Applied Biosystems), using the GeneScan-500 ROX^®^ Size Standard (Applied Biosystems), according to the manufacturer’s specifications. The real-time PCR reaction was performed using the PowerUp™ SYBR™ Green Master Mix (Applied Biosystems) intercalating dye and endogenous control. To calculate the amount of *SRY* gene, the *^ΔΔ^Ct* method was used. This method is based on a comparison of the level of gene expression in the test sample for endogenous control and a calibrator. The reaction was carried out on a StepONEPlus real-time PCR System (Applied Biosystems), and the results were calculated using the software attached to the instrument.

Before the analysis of gene expression by means of standard curves, a test of reaction efficiency of the tested gene and endogenous control were performed. The real-time PCR reaction was performed for the test gene and endogenous control on a 96-well plate, according to the protocols provided by the manufacturer (in triplicate for each sample), using a normal bull as the calibrator and the *GAPDH* gene as the internal control to normalize the data.

The reaction was performed in 13 µL reaction with 6.5 µL PowerUp™ SYBR™ Green Master Mix, a forward and reverse primer (0.8 µL, 10 µM) (Table 2) [6], a DNA template of 1.5 µL, and 4.2 µL Nuclease-free water. The qPCR thermal cycling condition was used, according to the protocols provided by the manufacturer.

## 3. Results

In 21 heifers, leucocyte chimerism determined by the 60, XX/60, XY karyotype was revealed—the proportion of the 60, XY male cell line in individual animals was in the range of 4% (calf 21) to 66% (calf 14) (Appendix A Table A1). In the case of two animals (No. 13 and 1), 2 and incomplete metaphase plates (respectively, 0.8 and 1.6%) with a chromosome similar to the morphology of the Y heterosome were revealed, which caused diagnostic doubts. In order to verify the suspicion of the XY line occurrence, the FISH experiment was carried out with the Y chromosome-specific molecular probe, but no hybridization signals identifying the Y chromosome were obtained. The absence of the Y heterosome testifies to the fact that the XY line was not borrowed from the male co-twin. On the basis of that cytomolecular analyses, the karyotype of both heifers was determined to be normal (60, XX).

Karyotype 60, XX was also identified in a third calf (No. 22) examined at the age of three days. Cytogenetic evaluation was verified by molecular methods.

In most cases, due to the early age of diagnosed heifers, veterinary examinations would have not been able to determine the degree of developmental disorders of the reproductive system. In contrast, in the case of two normal female calves (with karyotype 60, XX) originating from heterosexual twins, no visible characteristics of masculinization were found in the latter age. In one of them, at the age of 15 months, the pregnancy was diagnosed, and in the other one, at the age of 7 months, normal ovaries and uterus were revealed.

**Figure 1:**
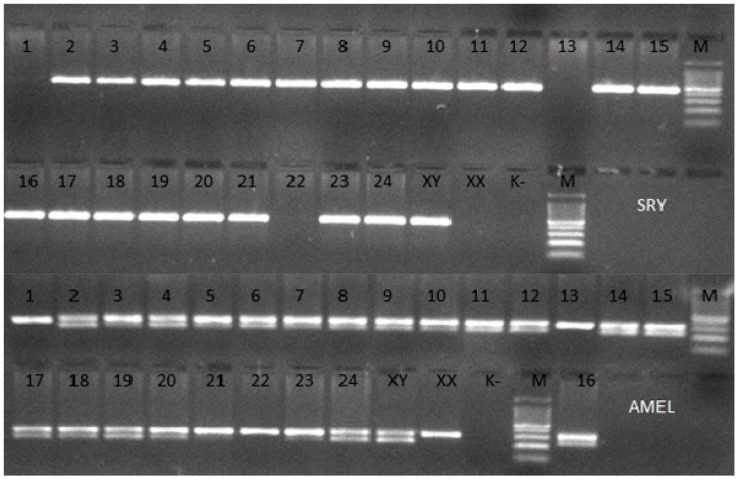
Agarose gel electrophoretic analysis of PCR of 24 different samples of bovine DNA with amplification of the *SRY* and *AMEL* genes. XY—Male, XX—Female, K^−^—negative control, M—100 bp DNA Marker.

The PCR amplification of the *SRY* and *AMEL* genes and agarose electrophoresis showed the absence of the *SRY* gene in 3 heifers (Figure 1). The *SRY* gene shortage is evidenced by the lack of a visible band and one visible band for the *AMEL* gene. The presence of the amplified fragment of the *SRY* gene in the test sample is proved by the occurrence of one band and two bands for the *AMEL* gene.

**Figure 2:**
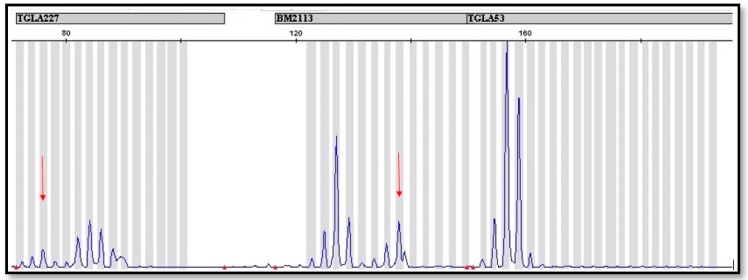
Fragment of microsatellite profile of a bovine with established leukocytic chimerism. The red arrows were used to mark additional alleles in the BM2113 and TGLA 227 locus, which indicate the occurrence of leukocytic chimerism.

In order to corroborate the molecular studies discussed above, the analysis of 12 microsatellite loci was performed. These analyses confirmed the results obtained by PCR reaction with the use of the *SRY* and *AMEL* genes. The STR analysis showed the presence of additional alleles in at least one locus, which indicates the occurrence of the second cell line (Figure 2). In two cases, a basic panel recommended by ISAG was not sufficient, so it was necessary to use the additional 8 markers to find a second line. Our observation suggests that the basic panel of 12 STR markers recommended by ISAG is insufficient to detect leukocyte chimerism if the percentage of the second line is low. In Appendix A Table A1, STR markers are listed, for which an additional allele was identified to indicate the possible occurrence of leukocytic chimerism in twins. It should be remembered that the microsatellite set recommended by ISAG is mainly used for parentage control as well as for population diversity analysis. In order to determine the presence of chimerism, the microsatellites on the Y chromosome should be used. The use of microsatellites recommended by ISAG is intended to compare this method with other available methods [19].

**Figure 3:**
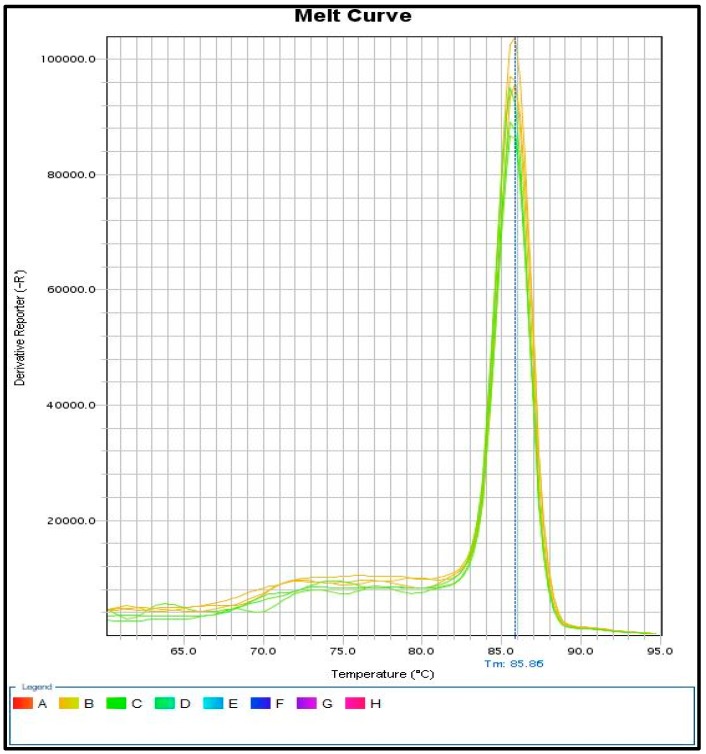
Results on melt curve analysis. One peak indicates the existence of one reaction product for a given gene, the correct design of primers, and the entire reaction system.

The identification of *SRY* gene fragments with real-time PCR gives even more accurate results compared to the methods described above. The use of fluorescent dyes that bind to a double-stranded PCR product gives the possibility to detect small amounts of the *SRY* gene in case of leukocytic chimerism. Importantly, a melt curve analysis should also be performed to check the homogeneity of the obtained PCR product (Figure 3).

Analyses carried out with the real-time PCR method indicate that there are differences in the level of the *SRY* gene between the examined heifers, which is related with a different percentage of the second line (60, XY). qPCR analysis confirmed the result obtained from different methods previously described. qPCR analysis with fluorescent dyes enables better, cheaper, and more accurate detection of leukocytic chimerism in young heifers. Early diagnosis of chimerism allows us to eliminate young infertile females from the herd. All the results, cytogenetic and molecular, have been shown in Appendix A Table A1.

## 4. Discussion

The phenomenon of freemartinism in females born from twins or multiple pregnancies carrying fetuses of different genders leads to a disturbed development of the reproductive system, including infertility [4,8]. The presence of two cell lines, differing in the heterosome set, is the effect of the formation of a shared blood system by the anastomoses or vascular connections between fetal membranes of co-twins before the beginning of the sexual differentiation of the fetuses [10]. Due to the fact that gonadal differentiation begins several days earlier in males than in females, sex-determining factors from the developing male gonad could be transported to the female fetus, causing malformation of female reproductive organs. The abnormalities of the female twin are caused by the anti-Mullerian hormone, which is responsible for the regression of the Müllerian structures in males during their normal sexual development and is involved in the morphological differentiation of the testes [11,12]. In heterosexual cattle twins, the regression of Müllerian ducts occurs between 50–80 days of fetal life, whereas the masculinization of the gonads and the development of the Wolffian ducts occur around the 90th day. Both twins exhibit high serum anti-Mullerian hormone concentrations, whereas the gonadal production of this hormone in females is very low [4,13]. The females with a XX/XY karyotype are sterile as an effect of extensive pathological changes in the reproductive system; however, the external genitalia in general are typically female. The observed changes include an enlarged clitoris, a small, blind-ending vagina, hypoplasia, or aplasia of the uterus. In addition, hypoplastic, sometimes masculinized, gonads may contain ovotesticular structures [14,15,16,17]. However, the ratio of XX to XY karyotypes in freemartins varies considerably from individual to individual and is not related to the degree of masculinization [1,8,9]. Previous cytogenetic analyses have shown that the frequency of XY cells in freemartins ranges from 2 to 99%, but an incorrect diagnosis may occur if the frequency of XY cells in a heterosexual female is less than 5% [6]. Additionally, in the case of our studies, in two animals, a slight proportion of the male cell line 60, XY was found (at the level of 0.8% and 1.6%), which resulted in the need to verify the initial diagnosis by using the FISH technique. The hybridizations performed in situ did not confirm the presence of the Y chromosome, and on that basis the karyotypes of both heifers were determined to be normal—60, XX (which was corroborated by molecular methods). The reporting in this paper of three cases of heifers with a normal karyotype 60, XX in a small population of 24 calves from heterosexual multiple pregnancies indicates that anastomoses did not occur in 12.5% of twins, and these potentially fertile heifers can be qualified for further breeding. Cytogenetic diagnosis of XX/XY chimerism is based on cell lines karyotyping and identification of heterosomes [18,19]. Such a diagnosis, requiring analysis of at least several hundred metaphases, is a laborious and difficult procedure, due to a large diploid chromosome number and their morphology. These traditional methods are often inaccurate in identifying sex chromosomes, so to be more precise, their determination needs the application of fluorescent in situ hybridization with X- and Y-specific molecular probes [16,20,21]. The FISH technique provides a fast and strict pinpointing of sex chromosomes and XX and XY cell lines in chimeric animals from different gender twin pregnancies an enables an estimation of the proportion of the co-twin line [22,23]. However, commercial FISH heterosome probes are only available for a few livestock species (including cattle), and the high costs prevents their use for routine cytogenetic analyses. Previous reports have shown that the proportion of XY cells in karyotypes of freemartins was not related with the degree of masculinization and inhibition of ovary or Müllerian duct development, whereas, currently, the diagnosis of freemartinism includes qualitative and quantitative detection of the *SRY* gene in the aspect of the effect on fertility of females originating from twin calves of different genders (6). In the situation when it is difficult to interpret unequivocally the results of cytomolecular analysis, like in case of two heifers with low percentage of leukocytes with the Y chromosome (0.8% and 1.6%) reported in our studies, it is suggested that the diagnosis should be performed with the use of both cytogenetic and molecular methods as a combined strategy that allows early, rapid detection of sex chromosome chimerism, saving time, effort, and financial outlays [9,23]. Therefore, most often for the diagnosis of freemartinism, a fast, sensitive, and less expensive PCR-based molecular analysis is used, especially routinely practiced in the parentage of (STRs) tests (multiplex PCR with the available commercial sets of STR markers distributed along the whole genome) [23]. In that case, it is recommended to analyze DNA isolated from blood cells and hair follicles with the aim to recognize one’s own and full-sib cell lines. However, this approach has some limitations because it is insensitive to the distinction between chimeras caused by the cell exchange between the opposite sex twins and twins of the same gender. Additionally, the analysis of X- and Y-linked gene (*SRY, AMELX/AMELY, ZFX/ZFY*) polymorphisms or anonymous markers assigned to the Y heterosome may also be used to detect the Y chromosome in XX/XY females [24,25,26,27]. However, if the Y-linked markers are only studied in blood cells, it does not facilitate to distinguish between the XX/XY chimerism and other disorders of sex development (DSD) [8]. The highest informativity and good accuracy for chimerism assessment offers the use of parentage QF-PCR(quantitative fluorescence polymerase chain reaction) - based STR screening [28,29,30]. Alternatively, more sensitive real-time PCR assays supplemented by STRs linked to the sex chromosomes can be applied [31]. According to these methods, different allele patterns in QF-PCR STR analysis in blood and hair samples can be explained by the coexistence of two different cell lines in the same animal. Therefore, the QF-PCR technique and real-time PCR assays could be suggested as a definitive diagnostic tool for determining cellular chimerism in several livestock species [6,25]. Furthermore, the most recent molecular method which can be considered as a valuable tool of identification of chimerism is genome-wide single nucleotide polymorphism (SNP) array analysis in various biological materials [32]. The SNP arrays use a combination of intensity (genomic dosage) and genotyping data from different tissues that provide high-resolution means to the differentiation of chimerism and mosaicism, since the additional presence of extra genotypes in the chimeras is readily detectable. Concretely, in the case of blood chimerism (associated with the occurrence of anastomoses), the mixed genetic profiles present in one blood sample can be easily recognized by the analysis of intensity parameters (copy number), which deviate from the expected values [33]. The use of modern cytogenetic and molecular techniques in the study of cell chimerism provides an increase in the diagnostic potential, which is necessary to determine freemartinism in very young female calves. A big advantage of using molecular methods is the low cost of analysis and the fact that the collected blood can be frozen, which makes it easier to transport the material to the laboratory, unlike in cytogenetic analyses where the test material is fresh blood collected in sterile tubes with heparin, which must be delivered to the laboratory within 24 h of collection. The precise and early identification of freemartinism can be the basis for formulating precise guidelines and selection recommendations regarding the reproductive performance of heifers born from heterosexual multiple pregnancies.

## Figures and Tables

**Table 1:** Primer sequences for amplification by PCR of the *SRY* and *AMEL* gene fragments.

Name	Sequence 5′–3′
SRY F	AAGGGGAGAACATGTTAGGGAGAG
SRY R	TTTGCAGGAGTGAATTGGTTATGA
AMEL F	CAGCCAAACCTCCCTCTGC
AMEL R	CCCGCTTGGTCTTGTCTGTTGC

**Table 2:** Primer sequences for PCR and real-time quantitative PCR.

Name	Sequence 5′–3′	Amplicon Size (bp)	TM (^°^C)
SRY F	GCCACAGAAATCGCTTCC	229	60
SRY R	CCGTGTAGCCAATGTTACCTT		
GADPH F	GTGAGAGACGGAACAGGAAGAA	110	60
GADPH R	ATGAGGGAAGACAGGACAAAGC		

## Appendix A

**Table A1:** Experimental material and results of cytogenetic and molecular analyses.

Animal *	Age [Months]	Cytogenetic Analysis		Molecular Analysis		
		60, XX (%)	60, XY (%)	SRY	AMEL Y	STR
1.	5	98.4 100	1.6 0	−	−	-
2.	8	67	37	+	+	TGLA 122, TGLA 227
3.	9.5	82	18	+	+	TGLA 227
4.	9	72	28	+	+	TGLA227
5.	11	56	44	+	+	TGLA53, ETH3
6.	9	81	19	+	+	TGLA227, ETH10
7.	3.5	56	44	+	+	TGLA227, ETH10, INRA23
8.	3	61	39	+	+	TGLA227, ETH10, INRA23
9.	2.5	65	35	+	+	TGLA53, SPS115, ETH225, AGLA293
10.	2	75	25	+	+	ETH3, BM1824
11.	2	71	39	+	+	TGLA53, TGLA126, ETH3
12.	0.5	64	36	+	+	TGLA126, ETH225
13.	12	99.2 100 ^†^	0.8 0	−	−	
14.	1	34	66	+	+	TGLA53, TGLA122, ETH10, CSSM66
15.	1	88	12	+	+	CSRM60 CSSM66, HUIJ177
16.	0.5	70	30	+	+	TGLA227, ETH225, ILSTS065
17.	1	48	52	+	+	TGLA 227, TGLA 53, INRA 23
18.	2	72	28	+	+	TGLA 227, TGLA 126, INRA 23
19.	2	84	16	+	+	ETH 10, TGLA 53, INRA 23
20.	0.5	57	43	+	+	TGLA 227, TGLA 122, INRA 23
21.	12	96	4	+	+	CSRM60
22.	3 days	100	0	−	−	-
23.	8	86	14	+	+	TGLA 53
24.	3	80	20	+	+	TGLA 53, ETH225

†—karyotype 60, XX determined with the use of the FISH technique; *—The number in the table corresponds to the number of the sample on the agarose gel.
